# Supplementary material for: Association between Prior Cytotoxic Therapy, Antecedent Hematologic Disorder, and Outcome after Allogeneic Hematopoietic Cell Transplantation in Adult Acute Myeloid Leukemia
Source: Cancers (Basel). 2023 Jan 5;15(2):352. doi: 10.3390/cancers15020352 (PMC9856876; doi:10.3390/cancers15020352)

Supplementary Materials: Association Between Prior Cytotoxic Therapy, Antecedent Hematologic Disorder, and Outcome after Allogeneic Hematopoietic Cell Transplantation in Adult Acute Myeloid Leukemia

Corentin Orvain, Eduardo Rodrìguez-Arbolì, Megan Othus, Brenda M. Sandmaier, H. Joachim Deeg, Frederick R. Appelbaum and Roland B. Walter

SUPPLEMENTARY TABLE S1. Primary disease and treatment-related characteristics in patients with therapy-related acute myeloid leukemia (n=115) and after antecedent hematologic disorder (n=125).

***Post cytotoxic therapy* (n=115)**	
**Previous condition (malignancy, auto-immune disease)**, n (%)	
Lymphoid hematologic malignancy	39 (34%)
Breast cancer	35 (30%)
Auto-immune disease	13 (11%)
Testicular cancer	5 (4%)
Thyroid cancer	5 (4%)
Sarcoma	4 (4%)
Uterine cancer	4 (4%)
Acute leukemia	3 (3%)
Prostate cancer	3 (3%)
Brain tumor	2 (2%)
Other solid tumors	2 (2%)
**Type of prior therapy**	
Chemotherapy, n (%)	98 (85%)
Radiation, n (%)	61 (53%)
Autologous HCT, n (%)	11 (10%)
Time interval from last treatment for previous condition to AML (IQR), years	3.3 (1.8 - 7.6)
***Antecedent hematologic disorder (AHD)* (n=125)**	
**Type**, n (%)	
MDS	97 (78%)
MPN	18 (14%)
CMML	10 (8%)
**Previously treated for another condition (malignancy, auto-immune disease)**, n (%)	20 (16%)
**Previously treated for AHD**, n (%)	50 (40%)
**Time interval from AHD to AML (IQR)**, years	0.66 (0.18 - 1.83)

Abbreviations: AHD, antecedent hematologic disorder; AML, acute myeloid leukemia; CMML, chronic myelomonocytic leukemia; HCT, hematopoietic cell transplantation; MDS, myelodysplastic syndrome, MPN, myeloproliferative neoplasm.

SUPPLEMENTARY TABLE S2. Pre-HCT demographic and clinical characteristics of study cohort (n=979), stratified according to disease status at diagnosis (de novo *vs.* post cytotoxic therapy *vs.* after antecedent hematologic disorder [AHD]), after considering as *de novo* AML, five patients with auto-immune disorders treated with methotrexate, mercaptopurine, and cyclophosphamide and 34 patients with AHD diagnosed less than three months before AML.

**Characteristic**	**All patients (n=979)**	***De novo* (n=798)**	**Post cytotoxic therapy (n=110)**	**AHD (n=71)**
**Age at HCT** (IQR), years	55 (42 - 64)	54 (41 - 62)	57 (48 - 66)	65 (58 - 69)
**Female gender**, n (%)	454 (46%)	367 (46%)	67 (61%)	20 (28%)
**WBC count at diagnosis** (IQR), G/l	8 (2 - 40)	9 (2 - 46)	5 (2 - 33)	3 (1 - 14)
**Cytogenetic risk (MRC)**, n (%)				
Favorable	72 (8%)	61 (8%)	11 (10%)	0
Intermediate	659 (70%)	545 (71%)	65 (61%)	49 (73%)
Adverse	213 (23%)	164 (21%)	31 (29%)	18 (27%)
**ELN-2017 risk classification**, n (%)				
Favorable	175 (18%)	148 (19%)	21 (20%)	6 (9%)
Intermediate	508 (54%)	422 (55%)	45 (42%)	41 (60%)
Adverse	264 (28%)	202 (26%)	41 (38%)	21 (31%)
**ELN-2022 risk classification**, n (%)				
Favorable	153 (16%)	130 (17%)	18 (17%)	5 (7%)
Intermediate	507 (54%)	425 (55%)	44 (41%)	38 (56%)
Adverse	286 (30%)	216 (28%)	45 (42%)	25 (37%)
**Time from last remission to HCT** (IQR), days	98 (69 - 146)	102 (71 - 148)	95 (69 - 130)	78 (57 - 112)
**Disease status at HCT**, n (%)				
First remission	747 (76%)	585 (73%)	101 (92%)	61 (86%)
Second remission	232 (24%)	213 (27%)	9 (8%)	10 (14%)
**MFC status before HCT**, n (%)				
MRD-negative	788 (80%)	660 (83%)	84 (76%)	44 (62%)
MRD-positive	191 (20%)	138 (17%)	26 (24%)	27 (38%)
**HCT-CI category at HCT**, n (%)				
Low	339 (35%)	309 (39%)	8 (7%)	22 (31%)
Intermediate	347 (35%)	292 (37%)	27 (25%)	28 (39%)
High	293 (30%)	197 (25%)	75 (68%)	21 (30%)
**Recovered peripheral blood counts before HCT**, n (%)	680 (69%)	568 (71%)	72 (65%)	40 (56%)
**Stem cell source**, n (%)				
BM	81 (8%)	72 (9%)	7 (6%)	2 (3%)
PBSC	766 (78%)	616 (77%)	88 (80%)	62 (87%)
Cord blood	132 (13%)	110 (14%)	15 (14%)	7 (10%)
**HLA matching**, n (%)				
Identical related donor	227 (23%)	180 (23%)	34 (31%)	13 (18%)
Matched unrelated donor	482 (49%)	387 (48%)	54 (49%)	41 (58%)
1-2 allele mismatch	101 (10%)	88 (11%)	4 (4%)	9 (13%)
Haplo-identical	37 (4%)	33 (4%)	3 (3%)	1 (1%)
Cord blood	132 (13%)	110 (14%)	15 (14%)	7 (10%)
**Conditioning regimen intensity**, n (%)				
MAC	583 (60%)	507 (64%)	48 (44%)	28 (39%)
Non-MAC	396 (40%)	291 (36%)	62 (56%)	43 (61%)

Abbreviations: BM, bone marrow; HCT, hematopoietic cell transplantation; HCT-CI, HCT comorbidity index; HLA, human leukocyte antigen; MAC, myeloablative conditioning; MFC, multiparameter flow cytometry; MRC, U.K. Medical Research Council; MRD, measurable residual disease; PBSC, peripheral blood stem cells; WBC, white blood cell count.

SUPPLEMENTARY FIGURE S1. Post-HCT outcomes for 990 adults with AML undergoing allogeneic HCT while in first or second morphologic remission, stratified by disease status at diagnosis (*de novo vs.* therapy-related *vs.* antecedent hematologic disorder) after considering as de novo AML, five patients with auto-immune disorders treated with methotrexate, mercaptopurine, and cyclophosphamide and 34 patients with AHD diagnosed less than three months before AML. (A) Non-relapse mortality, (B) relapse, (C) relapse-free survival, and (D) overall survival

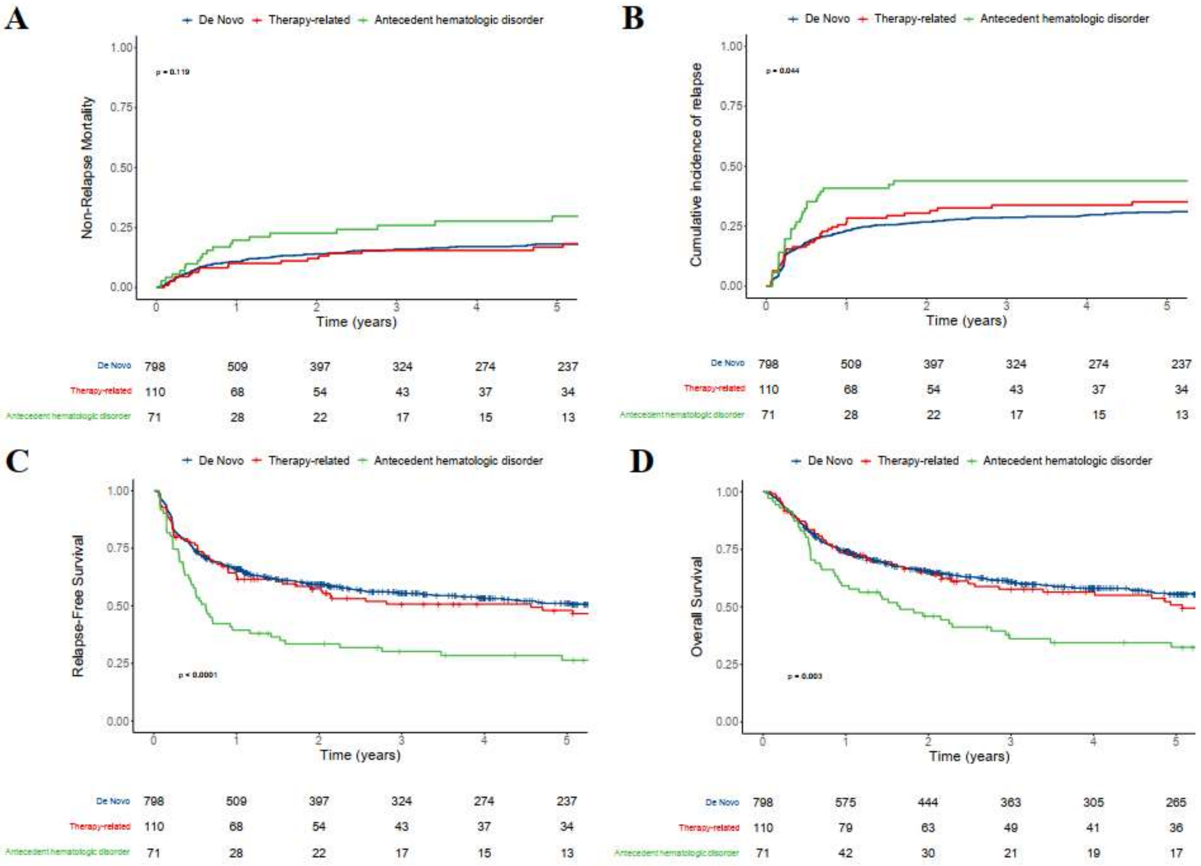

SUPPLEMENTARY FIGURE S2. Post-HCT outcomes for 125 adults with AHD AML undergoing allogeneic HCT while in first or second morphologic remission, stratified by type of disease (myelodysplastic syndrome [MDS] vs. chronic myelomonocytic leukemia [CMML] vs. myeloproliferative neoplasm [MPN]). (A) Non-relapse mortality, (B) relapse, (C) relapse-free survival, and (D) overall survival.

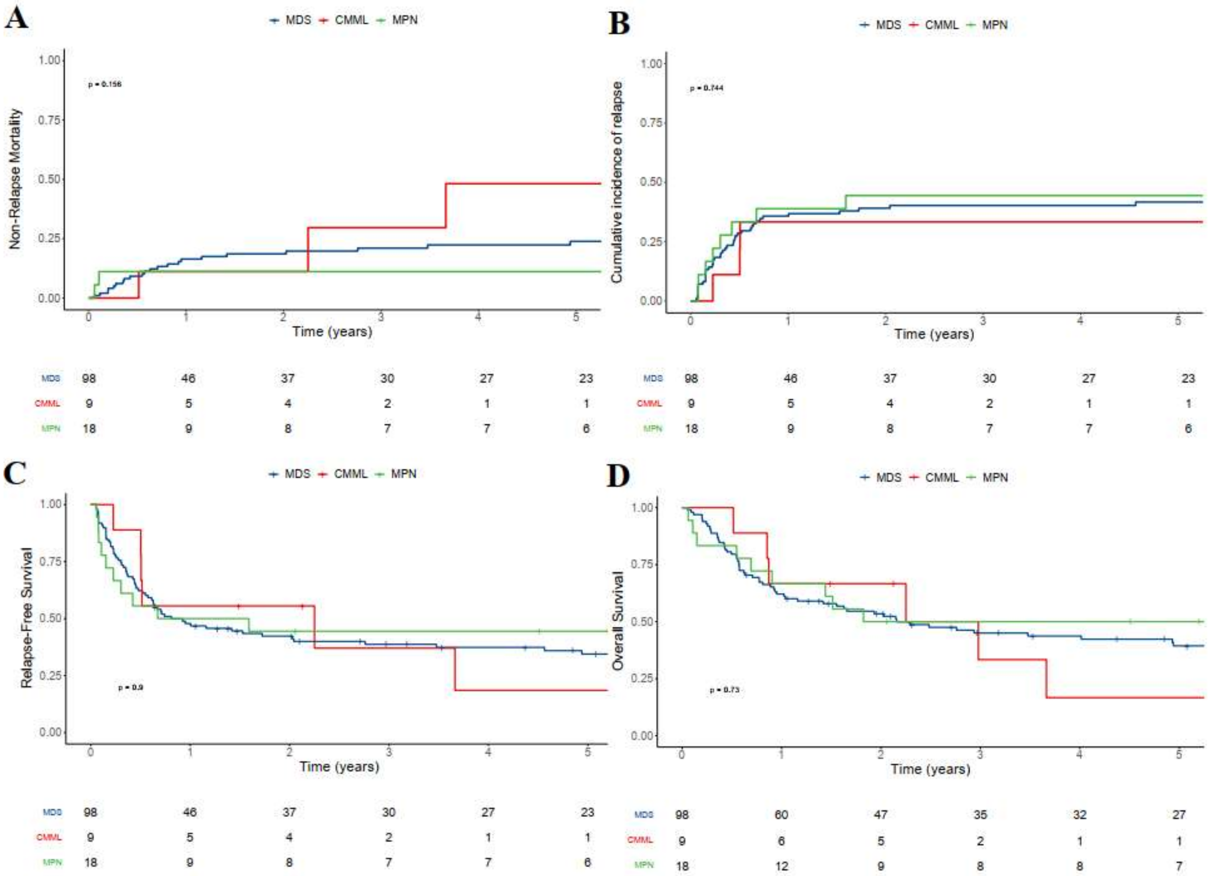

SUPPLEMENTARY FIGURE S3. Post-HCT outcomes for 125 adults with AHD AML undergoing allogeneic HCT while in first or second morphologic remission, stratified by prior treatment for AHD (yes *vs.* no). (A) Non-relapse mortality, (B) relapse, (C) relapse-free survival, and (D) overall survival

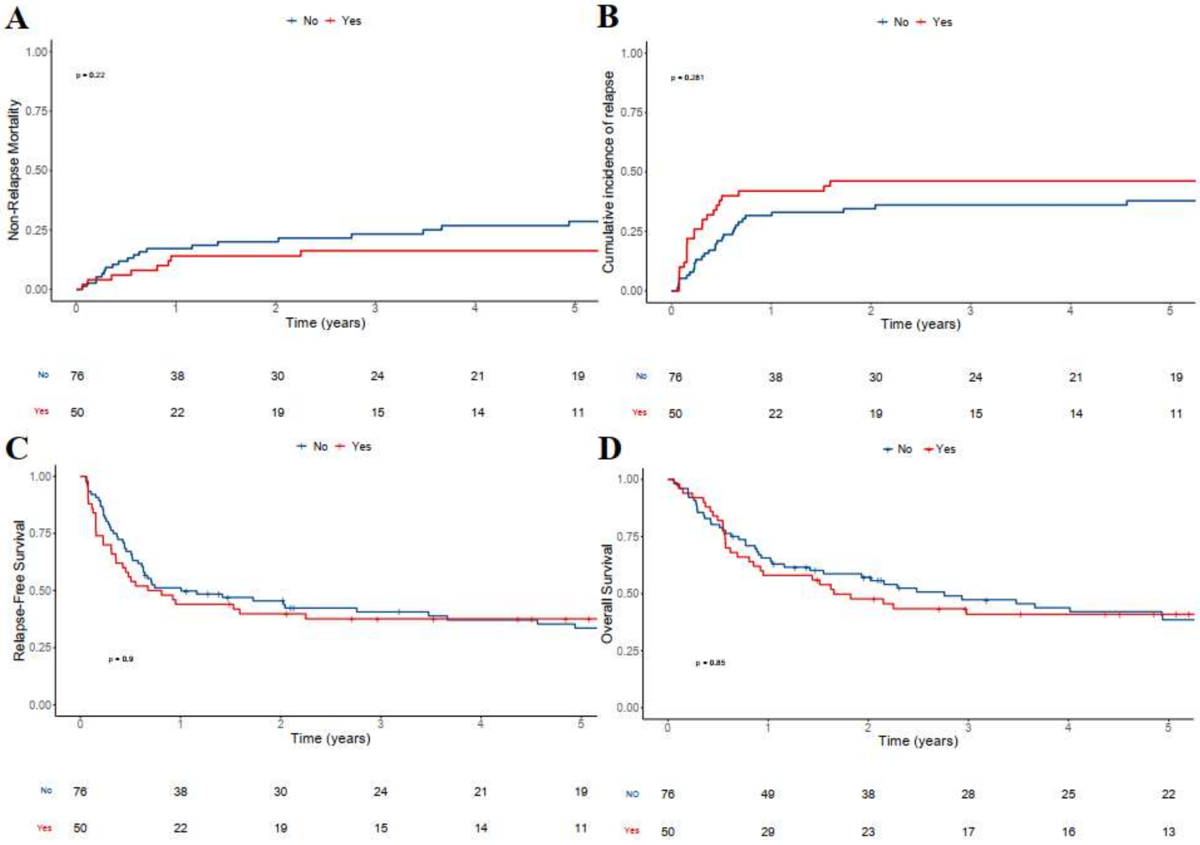

SUPPLEMENTARY FIGURE S4. Post-HCT outcomes for 143 adults with AML undergoing allogeneic HCT following reduced-intensity conditioning while in first or second morphologic remission, stratified by disease status at diagnosis (*de novo vs.* therapy-related *vs.* antecedent hematologic disorder). (A) Non-relapse mortality, (B) relapse, (C) relapse-free survival, and (D) overall survival.

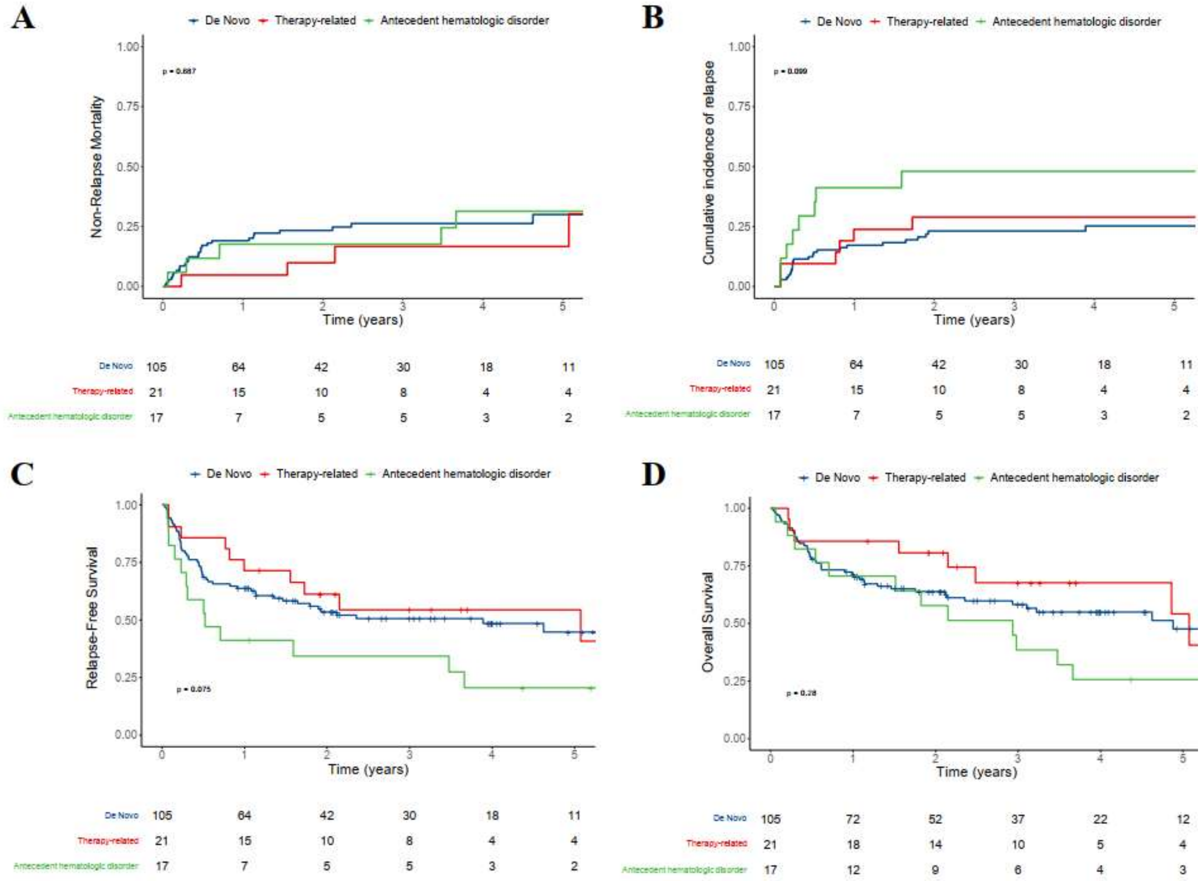

SUPPLEMENTARY FIGURE S5. Post-HCT outcomes for 253 adults with AML undergoing allogeneic HCT following non-myeloablative conditioning while in first or second morphologic remission, stratified by disease status at diagnosis (*de novo vs.* therapy-related *vs.* antecedent hematologic disorder). (A) Non-relapse mortality, (B) relapse, (C) relapse-free survival, and (D) overall survival.

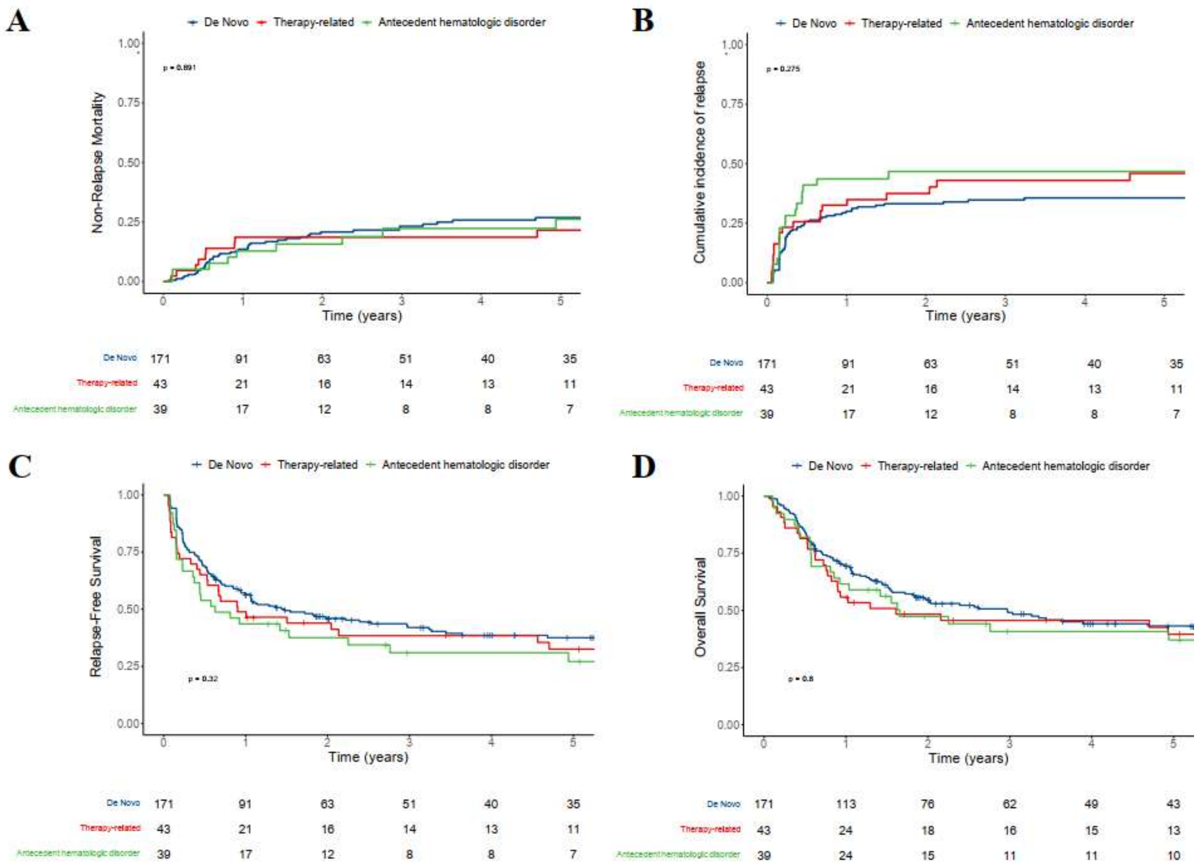

Supplement: Supplementary file 1 [file cancers-15-00352-s001.zip › cancers-2070780-supplementary.pdf]
